# Eco-friendly (green) synthesis of magnetically active gold nanoclusters

**DOI:** 10.1080/14686996.2017.1290492

**Published:** 2017-03-15

**Authors:** Naveen Reddy Kadasala, Lu Lin, Christopher Gilpin, Alexander Wei

**Affiliations:** ^a^ Department of Chemistry, Purdue University, West Lafayette, IN, USA

**Keywords:** Gold nanoparticles, magnetic properties, synthesis, composite materials, plasmon resonance, luminescence, sustainability, 40 Optical, magnetic and electronic device materials, 102 Porous / Nanoporous / Nanostructured materials, 103 Composites, 106 Metallic materials, 203 Magnetics / Spintronics / Superconductors, 204 Optics / Optical applications, 301 Chemical syntheses / processing, 308 Materials resources / recycling, 501 Chemical analyses, 503 TEM, STEM, SEM

## Abstract

Au–Fe_x_O_y_ composite nanoparticles (NPs) are of great technological interest due to their combined optical and magnetic properties. However, typical syntheses are neither simple nor ecologically friendly, creating a challenging situation for process scale-up. Here we describe conditions for preparing Au–Fe_x_O_y_ NPs in aqueous solutions and at ambient temperatures, without resorting to solvents or amphiphilic surfactants with poor sustainability profiles. These magnetic gold nanoclusters (MGNCs) are prepared in practical yields with average sizes slightly below 100 nm, and surface plasmon resonances that extend to near-infrared wavelengths, and sufficient magnetic moment (up to 6 emu g^–1^) to permit collection within minutes by handheld magnets. The MGNCs also produce significant photoluminescence when excited at 488 nm. Energy dispersive X-ray (EDX) analysis indicates a relatively even distribution of Fe within the MGNCs, as opposed to a central magnetic core.

## Graphical abstract

**Figure:**
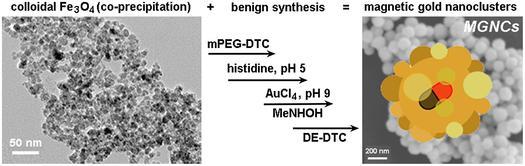

## 1. Introduction

Nanoparticles with hybrid magnetic–plasmonic properties are highly prized for applications in separations, biosensing, and nanomedicine [1–11]. These NPs are often comprised of a superparamagnetic iron-oxide core coated with a shell of metallic Au or Ag, which supports a localized plasmon resonance and a certain degree of chemical protection to the magnetic core. The metallic shells can be functionalized by a variety of surface chemistries, many of which enhance the dispersion and compatibility of NPs in biological media [12–19]. This has enabled the use of Au–Fe_x_O_y_ NPs for isolating and purifying biomolecular species like proteins and DNA, as multimodal contrast agents in biomedical imaging, and as photothermal agents for hyperthermia-mediated cancer therapies [3,8,10,11,20–26]. The optical properties of these nanocomposites can also be exploited for surface-enhanced Raman scattering (SERS) and other plasmon-enhanced processes, for the specific detection of trace analytes in aqueous media [27–29].

Despite their strong technological potential, nearly all syntheses of Au–Fe_x_O_y_ NPs have one or more steps that use nonpolar organic solvents, elevated temperatures, or a high concentration of non-biodegradable surfactants. All of these are negative factors from the perspective of sustainable manufacturing and lifecycle assessment, with significant burdens on the environment, energy consumption, and waste, which translates to higher production costs [30]. Sustainable manufacturing is practiced by chemical industries worldwide, with the intent of meeting the triple bottom-line goals of societal acceptance, cost-effective production, and environmental sustainability. Nano-manufacturing is based on similar principles, but must address inevitable tradeoffs between materials performance and sustainable production while still providing a net technological advantage. We thus seek alternative, greener methods for synthesizing Au–Fe_x_O_y_ NPs, with minimum concerns for environmental impact upon scaled production.

Recently, we reported a mild method of synthesizing magnetic gold nanoclusters (MGNCs) that is both simple and eco-friendly, and demonstrated their utility for detecting trace organic pollutants by SERS [29]. In this paper we describe an optimized and highly reproducible method for synthesizing MGNCs in aqueous alcohol, based on systematic adjustments in reagent concentrations and reaction conditions. Energy-dispersive X-ray (EDX) imaging by scanning electron microscopy (SEM) supports a heterogeneous distribution of Fe within MGNCs. The magnetization of the MGNCs can be as high as 6 emu g^–1^, sufficient for their precipitation by handheld magnets to enable applications in biomolecular separations.

## 2. Materials and methods

All materials were obtained from commercial sources and used as received, unless otherwise noted. Deionized water was obtained from an ultrafiltration system (Milli-Q, Millipore, Temecula, CA, USA) with a measured resistivity above 18 MΩ·cm, and passed through a 0.22-μm filter to remove particulate matter. CS_2_ was used as supplied and stored with minimum exposure to air.

Nanomaterials were characterized by transmission electron microscopy (TEM) using a Tecnai-T20 microscope (FEI, Hillsboro, OR, USA). TEM samples were prepared by floating carbon-coated grids on top of an aqueous NP dispersion for 30 min, followed by removal of the grid and drying in air for at least 60 min prior to analysis. Energy-dispersive X-ray (EDX) analysis by scanning transmission electron microscopy (STEM) was performed using a Tecnai G2 T20 microscope (FEI) equipped with a LaB6 filament and X-Max 80 silicon drift detector (Oxford, UK), with data collected by a high-angle annular dark field (HAADF) detector (Fischione, Export, PA, USA) and recorded by a 2 k × 2 k CCD imaging camera (Gatan, Pleasanton, CA, USA) using Inca software (ETAS, Stuttgart, Germany). Atomic absorption spectroscopy (AAS) was performed with a Perkin-Elmer (Waltham, MA, USA) 3110 spectrometer, using materials dissolved in aqua regia. Attenuated total reflectance-infrared (ATR-IR) analyses were performed on powder samples deposited on a ZnSe window, using a Nexus 670 spectrometer (Thermo, Waltham, MA, USA) flushed with N_2_ to remove atmospheric CO_2_ and moisture. Photoluminescence (PL) imaging was performed on air-dried samples with an Olympus (Tokyo, Japan) FV1000 laser scanning confocal microscope using a 10× confocal objective and 4.5× zoom lens, and three different laser lines with appropriate filters (excitation/emission = 488/505−525 nm; 543/560−620 nm; 635/655−755 nm).

Hydrodynamic size analysis of aqueous dispersions was performed by nanoparticle tracking analysis (NTA) using a Nanosight LM-10 (Malvern Instruments, Worcestershire, UK), with 405-nm laser excitation and distilled, particle-free water stored in polyethylene containers. Three tracking videos were collected per sample with a minimum of 2000 particle tracks per run, yielding hydrodynamic size (*d*_*h*_) values based on mode peak analysis. Inductively coupled plasma mass spectrometry (ICP-MS) was performed at the University of Illinois. Magnetic properties were measured at room temperature on neat powders using a MPMS-3 magnetometer (Quantum Design, San Diego, CA, USA) in vibrating sample magnetometer mode, with applied magnetic fields up to 10 kOe, and calibrated with colloidal γ-Fe_2_O_3_ [31]. Extinction spectra were obtained using a Cary-50 visible-near infrared spectrophotometer (Varian, Palo Alto, CA, USA) in transmission mode.

### 2.1. Synthesis and modification of colloidal iron oxide

Colloidal iron oxide (Fe_3_O_4_) was prepared by co-precipitation, using 648 mg of FeCl_3_ (4 mmol) and 398 mg of FeCl_2_∙4 H_2_O (2 mmol) dissolved in 5 ml of deaerated, deionized water, added dropwise to 15 ml of a 28% NH_4_OH solution over a period of 10 min in a glass test tube, while immersed in an ultrasonic cleaning bath. Colloidal Fe_3_O_4_ was formed immediately upon addition; care was taken to maintain anaerobic conditions during the dropwise addition of iron salts to the NH_4_OH solution. The reaction mixture was then removed from the ultrasonic bath and agitated for 2 min by vortex mixing to generate a homogeneous dispersion. Colloidal Fe_3_O_4_ was precipitated by applying an external handheld magnet along the walls of the reaction tube, then redispersed in deionized water. This process was repeated several times to remove weakly magnetized colloidal oxides. Final weights of magnetically active materials were obtained after drying the precipitated colloids in an oven, but were otherwise used as freshly prepared dispersions at a concentration of 8 mg ml^–1^.

To prepare mPEG-coated Fe_3_O_4_, 20 mg of 5-kDa mPEG-NH_2_ was dissolved in 1 ml of dry, deaerated methanol (4 mM) and stirred for 10 min, treated with one equivalent of CS_2_ (4 μmol) diluted in methanol and stirred for another 10 min, then treated with triethylamine (4 μmol) and stirred for 30 min at room temperature, resulting in mPEG-dithiocarbamate (DTC). Absorption spectroscopy confirmed DTC formation by the appearance of a doublet at 255 and 295 nm [32]. The freshly prepared mPEG-DTC solution was then combined with 1 ml of colloidal Fe_3_O_4_ dispersed in water (8 mg ml^–1^) and incubated at room temperature for 1 h. Aliquots were removed and air-dried for analytical characterization, but otherwise used as-prepared in the next step.

### 2.2. Synthesis of magnetic gold nanoclusters (MGNCs)

A 0.25-ml aliquot of freshly prepared dispersion of mPEG-DTC-treated colloidal Fe_3_O_4_ (1 mg) was added to 4 ml of an aqueous solution of L-histidine (1 mg ml^–1^), adjusted to pH 5–6 using 0.1 M HCl, then incubated at room temperature for 1 h. In a separate container, a 0.6-ml aliquot of 1% w/v HAuCl_4_ solution was diluted with 14.8 ml deionized water, adjusted to pH 9–10 using 5 M NaOH, then combined with the colloidal Fe_3_O_4_–histidine solution with vortex mixing and allowed to sit for 20 min. The reaction mixture (now pH 8–9) was treated with 20 mM *N*-methylhydroxylamine (NMH) to initiate reduction, added in five 0.2-ml portions with mixing every 5 min, with a noticeable change in color by the third addition. MGNCs were generated in significant quantities after 2 h at room temperature, and fully formed after 12 h. The reaction gradually increased in acidity to a final range of pH 6–7.

As-produced MGNCs were separated by selective precipitation using a handheld NdFeB magnet producing linear field gradients of 1–3 kG cm^–1^, followed 15–20 min later by decantation of magnetically unresponsive materials. The retentate was subjected to two more rounds of redispersion into water at twice the original volume with mild sonication, followed by magnetic precipitation, to yield MGNCs that were essentially devoid of non-magnetic gold NPs.

Excess Fe_3_O_4_ was removed from MGNCs by treating aqueous suspensions with a 0.5 M solution of diethanol-DTC in methanol (DE-DTC; final concentration 2 mM), prepared *in situ* from diethanolamine and CS_2_ [32]. In a typical cleansing procedure, 20 μl of 0.5 M DE-DTC was added to 5 ml of redispersed MGNCs (O.D. 0.4), followed by vortex mixing and mild sonication for several seconds, incubation for 1 h at room temperature, then two rounds of magnetic precipitation and redispersion in water.

## 3. Results and discussion

The co-precipitation of iron salts (commonly referred to as the Massart synthesis) is one of the simplest and most cost-effective approaches for preparing colloidal Fe_3_O_4_ [33]. However, this method is known to be sensitive to multiple reaction parameters, and often produces colloidal Fe_3_O_4_ with a broad size polydispersity, making it less suitable for NP syntheses requiring strict size control. On the other hand, the Massart synthesis is ideal from the perspective of sustainable materials chemistry, as it generates no organic waste or toxic byproducts. In our hands, co-precipitation typically yielded polycrystalline aggregates of Fe_3_O_4_ with domain sizes of 5–6 nm, and we have found these to be a reliable feedstock in the preparation of magnetic gold nanoclusters.

**Figure 1.**
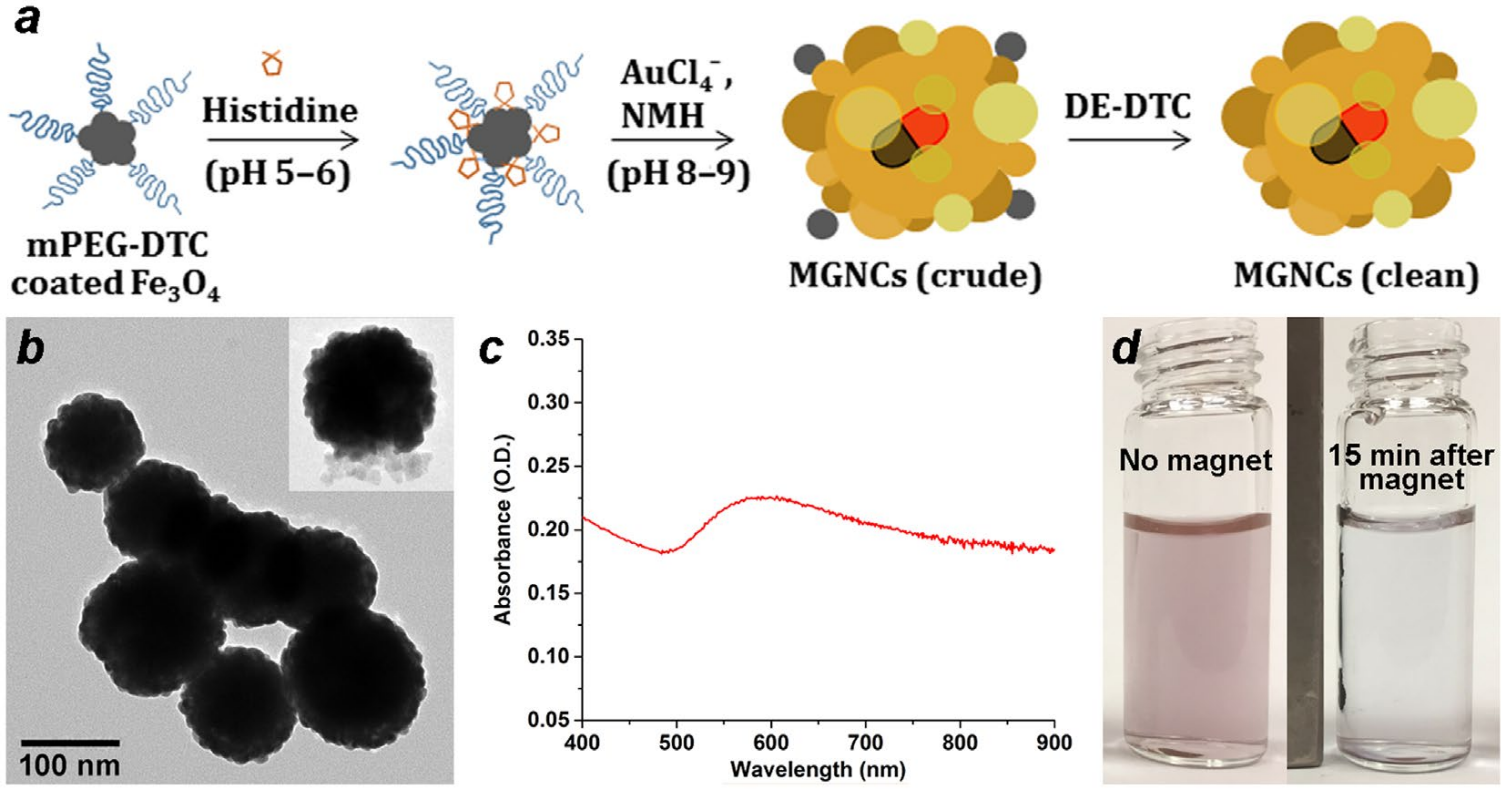
(a) Synthetic scheme for magnetic gold nanoclusters (MGNCs). (b) TEM image of clean MGNCs (*inset*: MGNC prior to DE-DTC treatment). (c) Optical extinction spectrum of MGNCs. (d) MGNCs before and after magnetic precipitation.

Preliminary studies on the electroless deposition of Au onto colloidal Fe_3_O_4_ were inspired by the work of Gao and coworkers, who showed that metallization can be induced on surfaces coated with poly-L-histidine via hydroxylamine reduction under basic conditions [11]. We find that the amino acid L-histidine also facilitates the reduction of HAuCl_4_ onto colloidal Fe_3_O_4_, but typically yields submicron composites in the absence of other surface-active agents (see below). After testing several different surface modifiers, we determined that treating colloidal Fe_3_O_4_ with mPEG-DTC (formed *in situ* from 5-kDa mPEG-NH_2_ and CS_2_ [34,35]) provided the best control in MGNC synthesis, with average cluster sizes close to 100 nm (Figure 1). Optical extinction spectroscopy indicated a broad plasmon resonance band with a maximum at 600 nm, but extending far into the near-infrared region. Previous electron diffraction analysis of MGNCs established the Au component to have an fcc structure [29].

**Figure 2.**
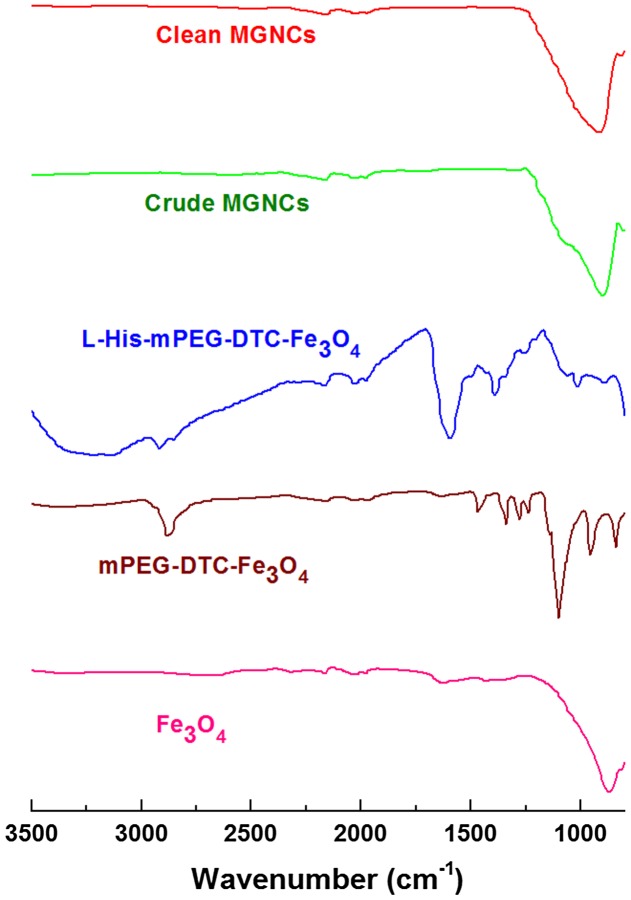
ATR-IR spectra acquired from pelleted samples after each step. Weak signals between 1900 and 2300 cm^−1^ are artifacts from instrumentation.

TEM images reveal that the MGNCs are often accompanied by significant amounts of extraneous inorganic material, presumed to be residual iron oxide. To remove this we turned to water-soluble dithiocarbamates (DTCs), which are well known for their chelation of transition-metal ions [36], and for their chemisorption onto metal surfaces [32,37]. DTCs have an especially high affinity for Fe^2+^ and Fe^3+^, suggesting utility as a digestive deferrating agent. This proved to be the case: treatment of as-prepared MGNC dispersions with DE-DTC, prepared *in situ* from a 2:1 mixture of diethanolamine and CS_2_ in methanol, removed all visible oxide from the MGNC surfaces and also from solution (Figure 1(b)). DE-DTC treatment does not introduce any notable changes to the optical or magnetic properties of the final dispersions, indicating that the MGNCs are uncompromised by the cleansing step. Likewise, attenuated total reflectance infrared (ATR-IR) spectroscopy indicates essentially no difference between MGNCs before and after treatment with DE-DTC (Figure 2).

The MGNCs are isotropic with morphologies varying from roughened spheres to raspberry-like clusters, with the latter being most common. TEM analysis indicates a range of grain sizes within MGNCs between 8 and 25 nm; again, treatment with DE-DTC did not appear to have any significant impact on either MGNC morphology or grain size.

**Figure 3.**
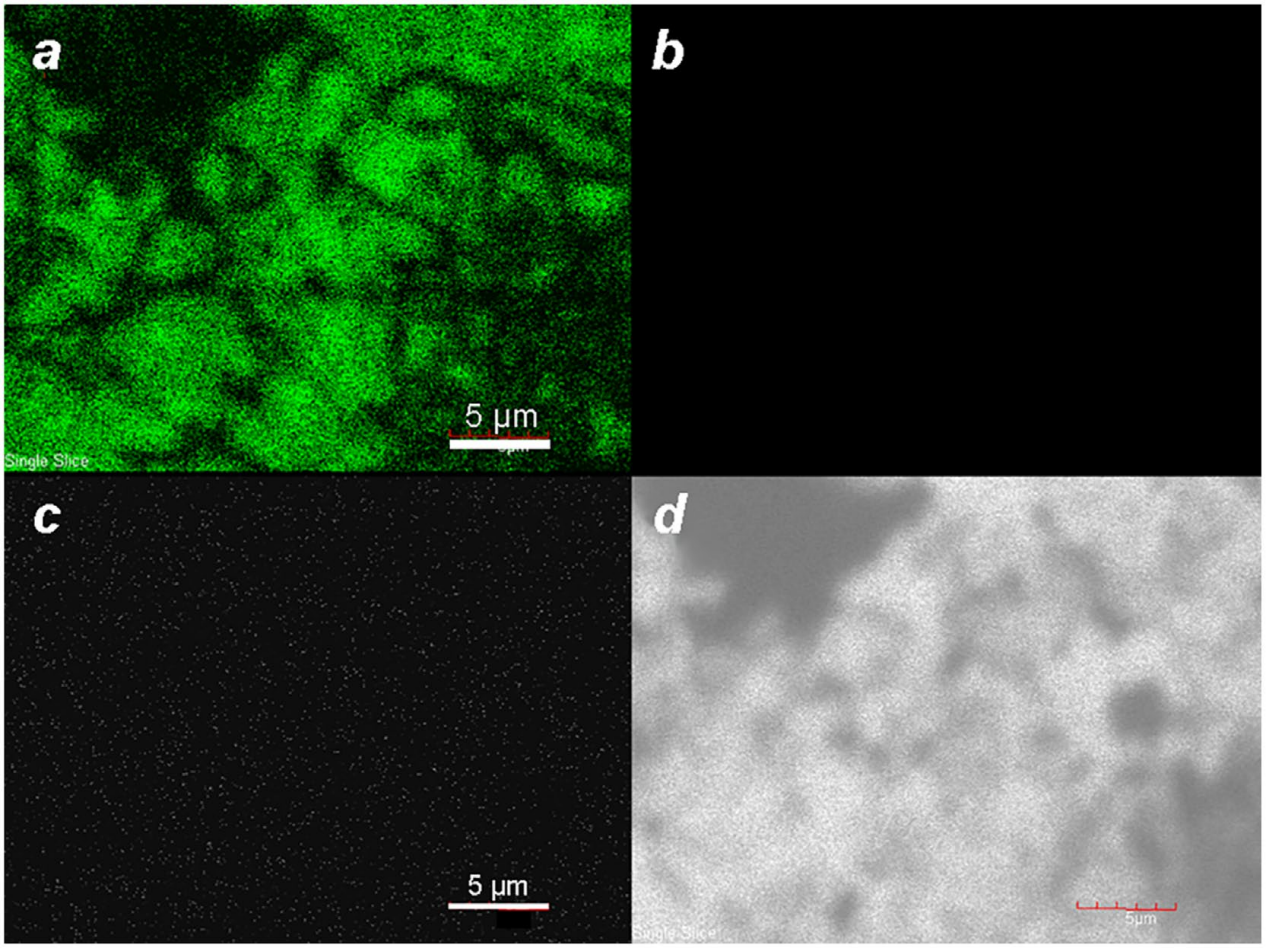
Laser scanning confocal fluorescence of MGNCs at different excitation wavelengths: (a) 488, (b) 546, (c) 647 nm. (d) Corresponding transmission image.

Earlier reports have shown that colloidal gold nanoparticles and nanoclusters can produce detectable levels of linear photoluminescence (PL) when excited at visible wavelengths, with emission intensities scaling roughly with particle volumes [38,41]. Analysis by laser scanning confocal microscopy (λ_ex_ 488 nm) yields a strong PL within the spectral window of 505–525 nm; however, no appreciable emissions are observed upon excitation at longer wavelengths (Figure 3). This is in accord with the reported mechanism for PL, which is produced by plasmon-enhanced emissions from excited *d*-band holes within gold nanostructures [38].

**Figure 4.**
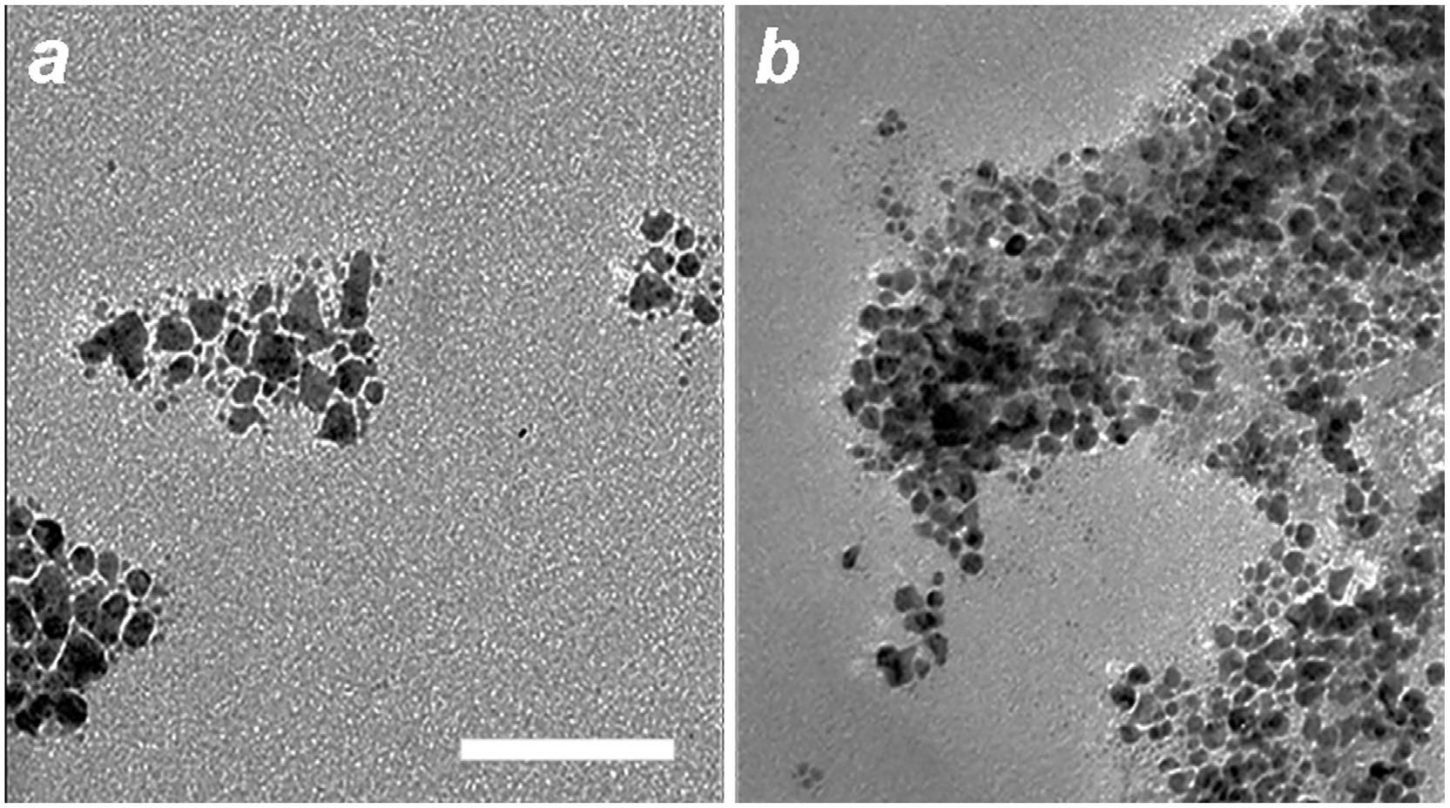
TEM images of (a) Au nanoparticles produced without L-histidine treatment of mPEG-DTC-coated Fe_3_O_4_ NPs; (b) aggregates produced without mPEG-DTC treatment of histidine-coated Fe_3_O_4_ NPs. Bar = 50 nm.

Control experiments illustrate the essential roles of both histidine and a peptizing agent for MGNC synthesis. Removal of either from the process results in the formation of small (10–12 nm) Au particles either loosely associated with iron-oxide nanoparticles, or embedded in a poorly dispersible oxide matrix (Figure 4). A weakly coordinating ligand such as histidine is necessary to encourage the adsorption of gold ions onto Fe_3_O_4_ surfaces, while a strongly anchored dispersant encourages the dissociation of iron-oxide particles from the parent aggregate. While the precise role of mPEG-DTC remains unclear, replacing it with low molecular-weight DTCs does not produce the desired MGNCs. We note that the reaction is sensitive to histidine concentration (0.2 g ml^–1^ after dilution); too much prevents the deposition of Au onto colloidal Fe_3_O_4_, resulting instead in smaller, non-magnetic NPs. The pH of the Fe_3_O_4_–histidine solution is also important, as histidine is cationic at pH 5–6 and can adsorb in that form onto Fe_3_O_4_ surfaces.

**Figure 5.**
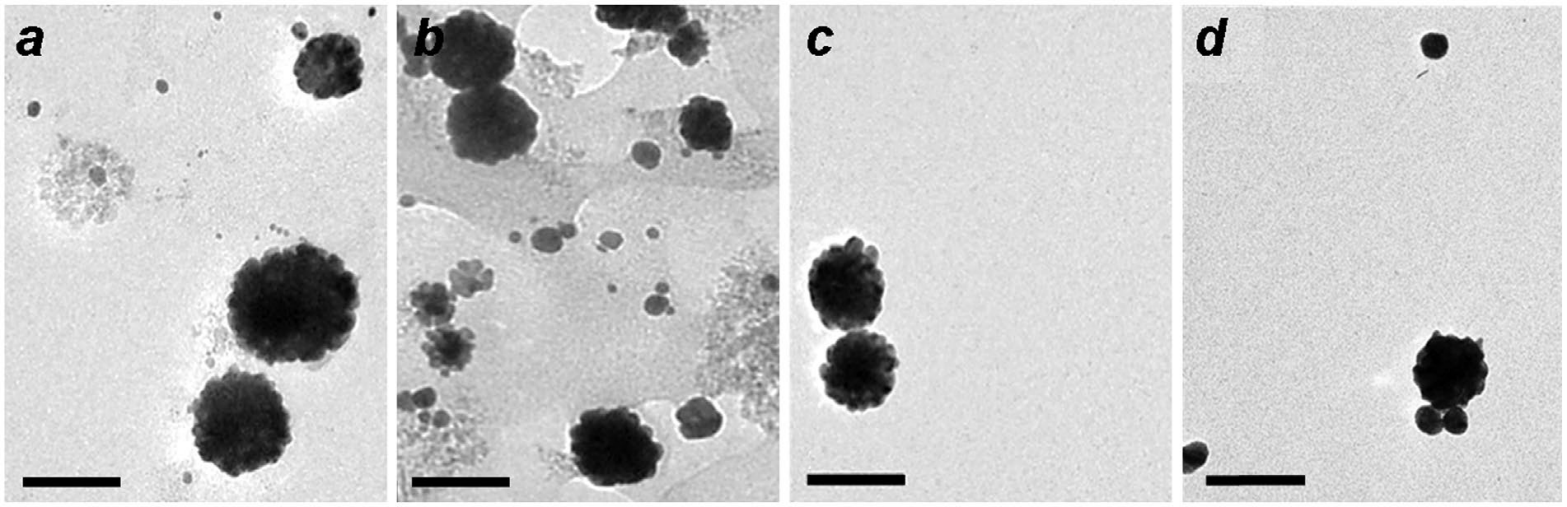
MGNCs prepared with different Au/Fe mole ratios: (a) 2; (b) 4; (c) 9; (d) 27. Bar = 100 nm.

The MGNCs are responsive to field gradients produced by handheld NdFeB magnets, enabling their facile separation and decantation from magnetically inactive gold NPs within a 15–20 min period. The exact composition of the iron oxide is unknown but likely to be a mixture of Fe_3_O_4_ and γ-Fe_2_O_3_, the latter produced upon oxidation by Au ions. Several experiments were conducted to optimize the incorporation of Fe_x_O_y_ in the MGNCs, in order to achieve higher magnetic moment. The mole ratio of gold to iron (Au/Fe) was varied to evaluate its effect on MGNC formation, size distribution, and magnetic response (Figure 5). A mole ratio of 6–9 produced MGNCs of relatively uniform size and shape, but lower Au/Fe ratios had variable effects on MGNC structures and also produced greater amounts of residual iron oxide, to the extent that their removal was problematic. Conversely, Au/Fe ratios well above 9 produce considerable amounts of non-magnetic colloidal Au (Figure 5(d)), which is readily determined by the residual reddish tint in solution after magnetic precipitation.

**Figure 6.**
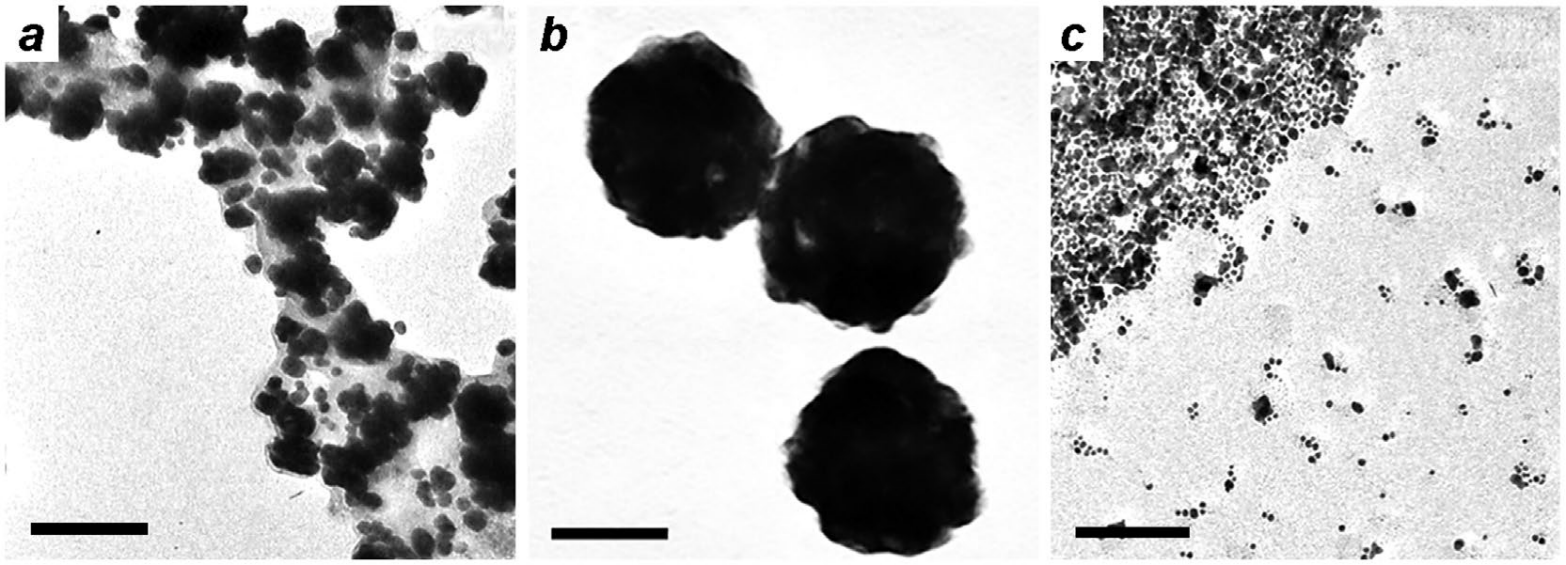
MGNCs synthesized using a fixed Au/Fe mole ratio of 9, with different initial pH values: (a) 6–8; (b) 8–9; (c) 9–10. Bar = 50 nm.

The effect of solution pH on MGNC formation was also evaluated at a fixed Au/Fe mole ratio of 9. Reductions performed under relatively acidic conditions (initial pH < 8) produces poorly dispersed MGNCs trapped in a matrix of amorphous iron oxide, whereas reactions performed at an initial pH of 8–9 produces well-dispersed MGNCs with a narrower size and shape polydispersity (Figure 6). Reactions performed at pH > 9 generates very small particles and with poor dispersion stability.

**Figure 7.**
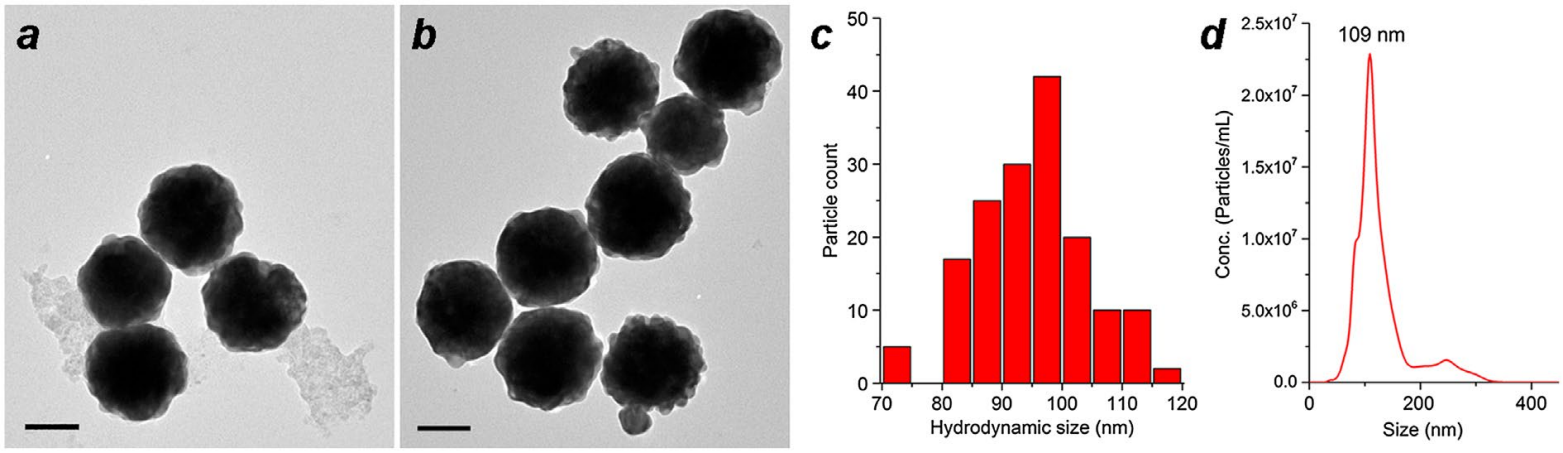
(a, b) TEM images of MGNCs prepared on a 20-ml scale, before and after cleansing with DE-DTC. (c) Size histogram of DTC-treated MGNCs by TEM image analysis (*d*_*av*_ = 95 nm). (d) Hydrodynamic analysis of MGNCs by NTA (*d*_*h*_ = 109 nm; standard error = 3 nm); Scale bar = 50 nm.

All reactions up to this point were performed on a small (1–5 ml) scale for the systematic evaluation of reaction conditions. To determine whether the optimized MGNC synthesis could be reproduced on a larger scale, the reaction was performed in bigger tubes (ca. 20 ml final volume; see Materials and methods), which produced MGNCs very similar in size as those made on a 1–5 ml scale (Figure 7). The dry weight of MGNCs following removal of residual iron oxide (see below) was estimated to be 10 mg (42% yield). The reproducibility rate is 50%, which is acceptable given the heterogeneous nature of the colloidal Fe_3_O_4_ feedstock. Refinement of co-precipitation conditions may further improve the reproducibility of Au deposition onto colloidal Fe_3_O_4_ surfaces.

The size distribution of DE-DTC-treated MGNCs prepared on a 20-ml scale was characterized by TEM image analysis (*d* = 95 ± 9 nm; *N* = 161) and by nanoparticle tracking analysis (NTA; *d*_*h*_ from 3 runs = 109 nm). The size analysis outcomes are similar and validate the accuracy of the latter method, in accord with other studies showing similar matches between TEM and NTA [35,39].

Elemental analysis was performed using ICP-MS and AAS to determine the percentage and distribution of iron within the DTC-cleansed MGNCs. Despite an initial Au/Fe ratio of 9 in the reaction mixture, ICP-MS and AAS both show MGNCs to have much higher mole ratios (Au/Fe = 35.5 and 26.8, respectively), meaning that most of the starting Fe_3_O_4_ is not incorporated into the product. We presume that (a) the reduction of Au onto histidine-coated Fe_3_O_4_ is inefficient, relative to its autocatalytic deposition on Au islands formed subsequently, and (b) much iron oxide is lost by the speciation of dissolved Fe upon addition of AuCl_4_, which is a strong oxidant and a source of halide counterions.

**Figure 8.**
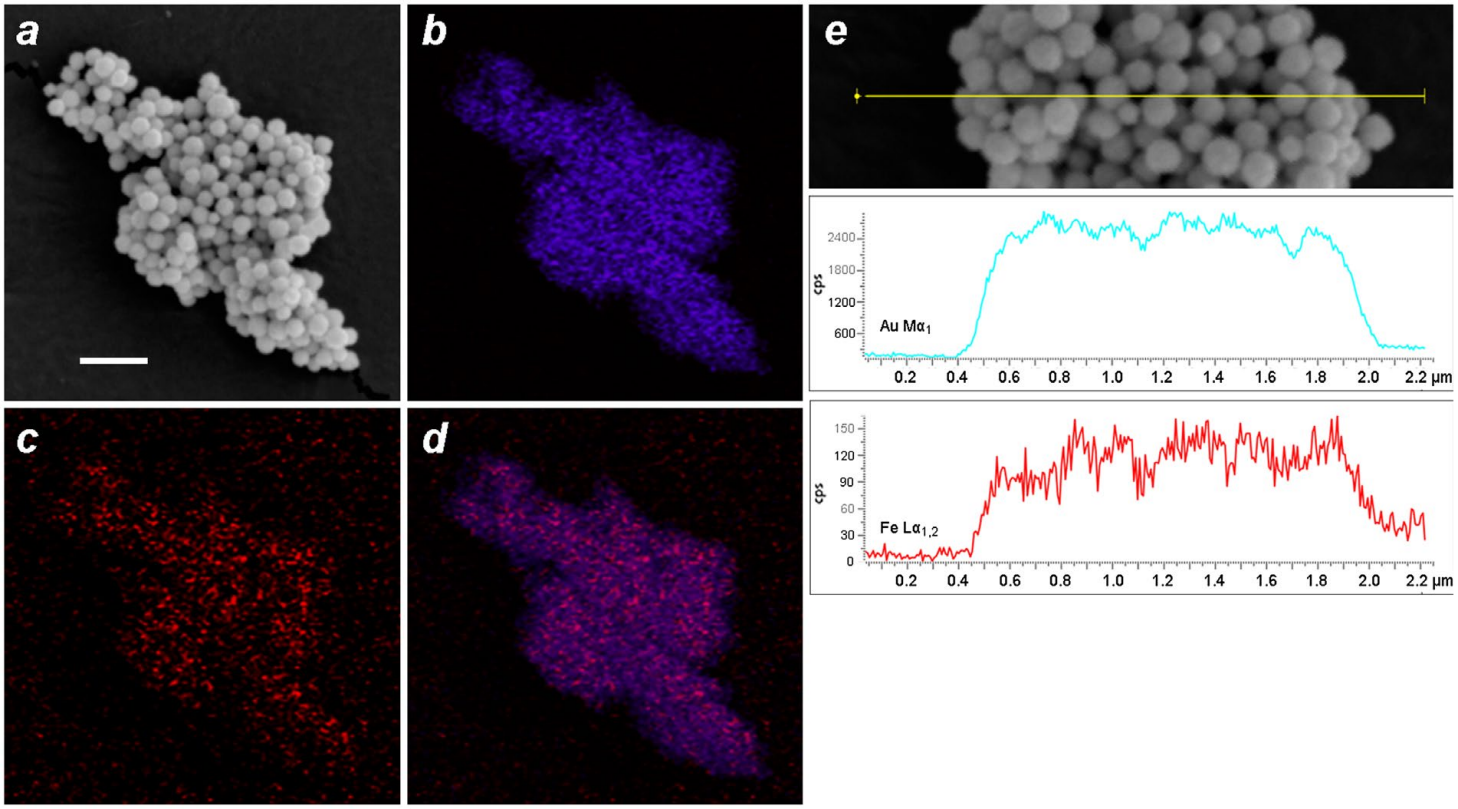
EDX–SEM data for DTC-cleansed MGNCs. (a) Backscattering image (bar = 500 nm), (b–d) elemental mapping for Au (Mα_1_: 2,123 eV), Fe (Lα_1,2_: 705 eV), and merged Au/Fe respectively. (e) Linescan across MGNCs, confirming the colocalization of Au and Fe.

**Figure 9.**
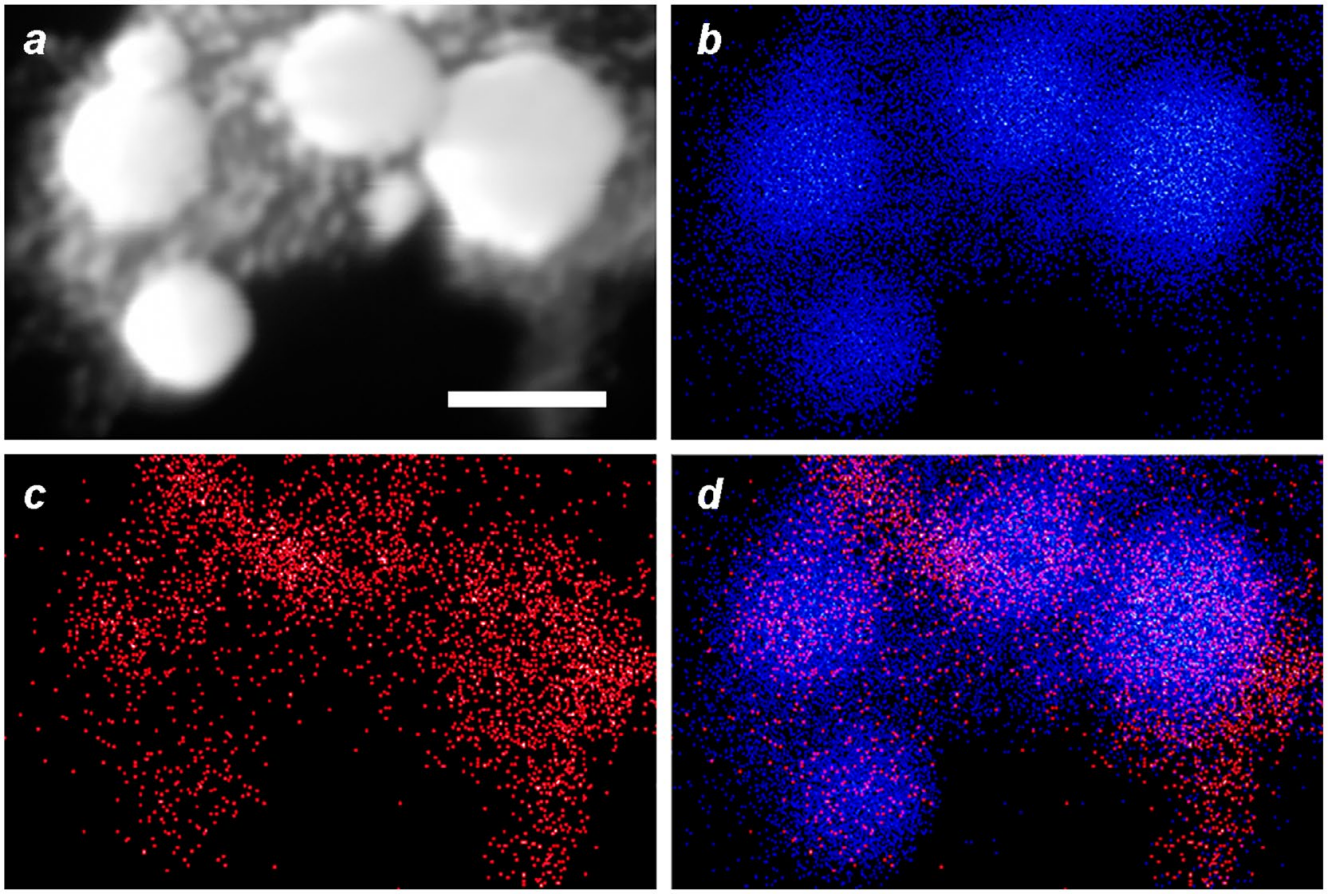
EDX data for MGNCs in HAADF–STEM mode, prior to cleansing by DE-DTC. (a) HAADF–STEM image (bar = 100 nm), (b–d) elemental maps for Au, Fe, and merged Au/Fe respectively. The distribution of Fe within MGNCs is relatively even.

EDX imaging was performed to determine the spatial distribution of Fe within the MGNCs. Low-resolution EDX–SEM imaging of MGNCs cleansed with DE-DTC confirmed colocalization of iron and gold within the MGNCs, but with a low content of Fe (Figure 8). To determine whether a core–shell morphology might be present, a higher resolution analysis was performed on individual MGNCs in HAADF–STEM mode, prior to DE-DTC cleaning (Figure 9). These images indicate a heterogeneous distribution of Fe with significant signal intensities within the MGNCs, but offer no evidence of a well-defined core-shell morphology.

We postulate that Fe_3_O_4_ is entrapped by the rapid growth and coalescence of Au domains, resulting in composite Au nanoparticles containing clusters or veins of superparamagnetic iron oxide. We note that a similar deposition mechanism has been described in the formation of Au–Fe_3_O_4_ ‘nanoroses’, in which 5-nm Fe_3_O_4_ particles were used to nucleate the deposition and growth of Au clusters [22]. Interestingly, EDX analyses from that study were also unable to confirm the existence of well-defined iron-oxide cores.

**Figure 10.**
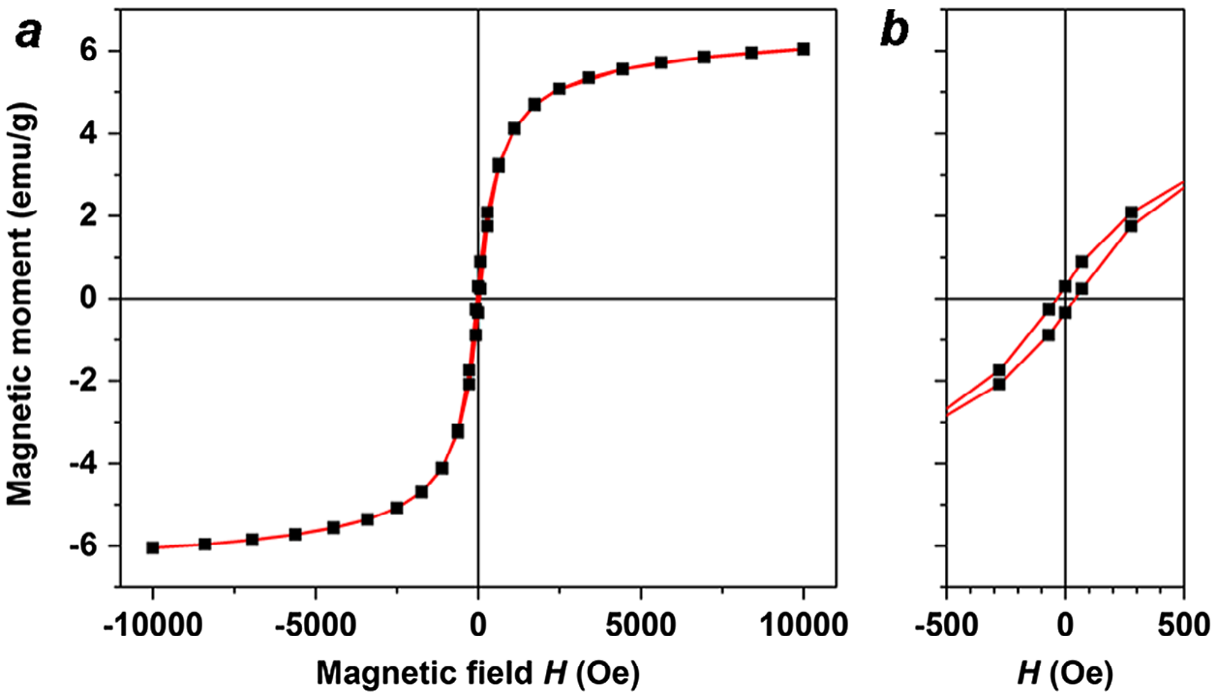
(a) Magnetization curve of MGNCs in powder form, taken at room temperature; (b) expansion of low-field region.

Despite the low Fe concentration, the MGNCs have sufficient moment to respond to local field gradients produced by rare-earth magnets (Figure 1(d)). The magnetic properties of MGNCs were characterized in powder form using a superconducting quantum interference device (SQUID) in vibrating sample magnetometry mode, with a field sweep of ± 10 kOe. Several MGNC samples were measured, yielding saturation magnetization (*M*_*S*_) values between 1.6 and 6.0 emu g^–1^ (Figure 10(a)). This variability in magnetization is to be expected, given the stochastic nature of Fe_3_O_4_ incorporation into the MGNCs during electroless deposition. On the one hand, this variability may limit the use of MGNCs in situations that require well-defined moment-to-mass ratios; on the other, many applications can tolerate a significant variation in magnetic moment, particularly those that require a magnetomotive function. It is worth mentioning that MGNCs are weakly ferromagnetic at room temperature, with *H*_C_ on the order of 35 Oe (Figure 10(b)). The source of coercivity in MGNCs remains to be determined; however, we note that remanent magnetization has been observed in other magnetic gold NPs, including those without any ferromagnetic elements [40].

## 4. Conclusions

A practical, eco-friendly synthesis of magnetic gold nanoclusters can be performed in aqueous alcohol without the use of harsh reagents, amphiphilic surfactants, or phase transfer from organic solvents. The final particles are produced with a narrow size distribution close to 100 nm, absorb strongly at NIR wavelengths, and can be collected within minutes using handheld magnets. EDX-SEM imaging supports the colocalization of Fe and Au within individual MGNCs. The MGNCs produce significant photoluminescence when excited at 488 nm. Lastly, ongoing studies have confirmed that MGNCs are highly biocompatible with cellular systems, and can thus be used to support a variety of bionanotechnology applications.

## Disclosure statement

No potential conflict of interest was reported by the authors.

## Funding

This work was supported by the Division of Civil, Mechanical and Manufacturing Innovation [grant number 1344654] and National Cancer Institute [grant number P30 CA023168].
